# Nutritional resuscitation of children with disordered eating.

**DOI:** 10.1186/2050-2974-3-S1-O65

**Published:** 2015-11-23

**Authors:** Lyza Norton, Laura Owens

**Affiliations:** 1 Gold Coast University Hospital, Queensland, Australia

## 

Medically compromised patients with disordered eating are admitted to the childrens ward of the Gold Coast University Hospital following a treatment protocol, including immediate insertion of nasogastic tube and commencement of enteral feeding, which is independent of admitting diagnosis, including anorexia nervosa, eating disorder not otherwise specified, anxiety and mood disorders.

Data has been collected and analysed over a 12 month period to include the diagnosis, BMI Z-scores corresponding to severity of malnutrition on admission, weight gained over admission, length of stay and follow up plans including readmission rates.

This presentation highlights the importance clear admission criteria and immediate nutritional resuscitation for the spectrum of disordered eating diagnoses and its effect on rectifying malnutrition, reducing readmission rates and improving the health outcomes for this population.

